# The structure of well-being: a single underlying factor with genetic and environmental influences

**DOI:** 10.1007/s11136-023-03437-7

**Published:** 2023-05-20

**Authors:** Ludvig Daae Bjørndal, Ragnhild Bang Nes, Nikolai Czajkowski, Espen Røysamb

**Affiliations:** 1 Department of Psychology, PROMENTA Research Center, University of Oslo, PO Box 1094, 0317 Oslo, Norway; 2 Division of Mental and Physical Health, Norwegian Institute of Public Health, Oslo, Norway; 3 Department of Philosophy, Classics, and History of Arts and Ideas, University of Oslo, Oslo, Norway

**Keywords:** Well-being, Genetics, Environment, Happiness, Well-being factors, h-factor

## Abstract

**Purpose:**

The structure of well-being has been debated for millennia. Dominant conceptualisations, such as the hedonic and eudaimonic models, emphasise different constituents of the well-being construct. Some previous studies have suggested that the underlying structure of well-being may consist of one or a few general well-being factors. We conducted three studies to advance knowledge on the structure of well-being comprising more than 21,500 individuals, including a genetically informative twin sample.

**Methods:**

In Study 1, we used hierarchical exploratory factor analysis to identify well-being factors in a population-based sample of Norwegian adults. In Study 2, we used confirmatory factor analysis to examine the model fit of the identified factor model in an independent sample. In Study 3, we used biometric models to examine genetic and environmental influences on general well-being factors.

**Results:**

We identified six well-being factors which all loaded on a single higher-order factor. This higher-order factor may represent a general “happiness factor”, i.e. an *h-factor*, akin to the *p-factor* in psychopathology research. The identified factor model had excellent fit in an independent sample. All well-being factors showed moderate genetic and substantial non-shared environmental influence, with heritability estimates ranging from 26% to 40%. Heritability was highest for the higher-order general happiness factor.

**Conclusion:**

Our findings yield novel insights into the structure of well-being and genetic and environmental influences on general well-being factors, with implications for well-being and mental health research, including genetically informative studies.

**Supplementary Information:**

The online version contains supplementary material available at 10.1007/s11136-023-03437-7.

## Introduction

What is happiness? This question has been asked for millennia and is an important topic in many philosophical and religious traditions. For instance, Aristotle’s writings on eudaimonia represent an early, yet still influential, inquiry into the structure of well-being [1]. Well-being is also an important theme in stoic [2] and Confucian (R. [3] philosophy. These different traditions emphasise different aspects of well-being—yet all are concerned with the question of what well-being encompasses.

The structure of well-being is also a topic of debate in research. Gallagher et al. [4] broadly distinguish between hedonic, eudaimonic, and social well-being models. A prominent example of a hedonic model is the subjective well-being (SWB) model, comprising pleasant affect, (absence of) unpleasant affect, and life satisfaction [5–7]. Life satisfaction has also been conceptualised as a core indicator of evaluative well-being [8]. The eudaimonic Psychological Well-being (PWB) model was proposed by Ryff [9]. It includes six components: self-acceptance, positive relations with others, autonomy, environmental mastery, a sense of purpose in life, and personal growth. Both frameworks have spawned much research [6, 10]. Other models emphasise the importance of social aspects of well-being (e.g. see Ref [11].

More recent approaches have attempted to integrate components from theoretically distinct well-being models. For instance, Keyes [12] included aspects of hedonic, eudaimonic, and social well-being in the ‘flourishing mental health’ model. Another recent model included five components: positive emotion, engagement, relationships, meaning, and accomplishment (PERMA) [13, 14]. Integrative efforts are supported by studies showing that hedonic and eudaimonic well-being are highly correlated (e.g. [15–17], even though they are theoretically assumed to capture different aspects of well-being.

Recent years have seen a development towards a hierarchical framework in well-being research, mirroring other areas of psychological science [18]. Several studies have found that a few general factors largely explain variance in well-being items in hierarchical and bifactor models (e.g. see Refs [4, 15, 16, 19–22] and that models with a single factor may show similarly good or superior fit to the data [23–25, 27]. These studies converge to suggest that the underlying structure of well-being may consist of one or a few general well-being factors. Recent studies have also examined well-being structure using a network psychometric approach (e.g. see Refs [28, 29].

Several questions pertaining to a hierarchical framework for well-being remain unresolved. First, there are inconsistencies in the numbers of identified well-being factors across studies. Second, few studies have used items from multiple well-being measures and conceptual frameworks. Third, many studies suffer from small sample sizes and low statistical power. Fourth, most studies have tested pre-defined theoretical models using confirmatory factor analysis (CFA). Combining CFA with data-driven approaches, such as exploratory factor analysis (EFA), could yield new insights into well-being factors. This has only been done in a small number of studies (e.g. see Ref [17, 20, 30].

Elucidating the structure of general well-being factors could have implications for theoretical models and development. Furthermore, well-being is measured in a myriad of ways [18, 31, 32]. Heterogeneous and unsystematic conceptualisations of well-being pose a challenge for well-being research, as it may limit robustness, replicability, and comparability of findings across studies. Previous work has also highlighted the importance of sound well-being measurement for public policy (e.g. see Ref [33, 34]. Promoting population well-being is a Sustainable Development Goal [35], and evaluating developments in well-being, for instance in response to public policies, requires comprehensive measuring of the construct.

Well-being and life satisfaction are influenced by genetics to a moderate extent, with heritability estimates in the range of 30–40%, which leaves 60–70% of variance in well-being accounted for by environmental influences [36, 37]. A few studies have examined genetic and environmental effects on latent well-being factors and reported higher heritability estimates, such as 48% for a ‘well-being factor’ comprising multiple subfactors [20] and 72% for ‘mental well-being’ comprising emotional, social, and psychological well-being [38]. The genetic correlation (i.e. the genetic overlap) across well-being aspects may also be substantial [20, 39–41].

However, few studies have investigated the genetic and environmental architecture of general well-being factors and used items measuring multiple well-being dimensions. Gaining a better understanding of influences on well-being factors could have implications for current understanding of well-being and future studies, such as genomic studies aiming to identify specific genetic variants associated with well-being.

In the current study, we seek to advance knowledge on the structure of well-being using three large samples. The items cover several dimensions, including hedonic, eudaimonic, and social aspects of well-being. Our primary aims are threefold:

1. In Study 1, examine the hierarchical structure of well-being in a large, population-based sample of Norwegian adults (*N* = 17,417).
2. In Study 2, test the fit of the model identified in Study 1 in an independent sample of Norwegian adults (*N* = 2125).
3. In Study 3, estimate genetic and environmental influences on well-being factors in a population-based sample of adult twins (*N* = 1987).

## Methods

### Participants

We used data from three Norwegian studies. The sample size comprised 21,529 individuals in total.

#### Quality of life survey 2020

The nationwide Quality of Life Survey 2020 (QoL 2020) was conducted by Statistics Norway in March 2020. A random sample of 40,000 individuals was invited to participate and 17,417 responded (44%). In total, 10% of participants were aged 18–24 years, 31% were 25–44 years, 42% were 45–66 years, and 17% were 67 years and older. 51% of participants identified as female.

#### Quality of life survey in Hallingdal 2019

The Quality of Life Survey in Hallingdal 2019 (QoL 2019) was conducted by Statistics Norway in Hallingdal in Norway. A sample of 4000 adults was invited to participate and 2125 responded (53%). Data collection was conducted in March and April 2019. The sample was drawn randomly but stratified based on population size within the six individual municipalities. In total, 9% of participants were aged 18–24 years, 29% were 25–44 years, 45% were 45 to 66 years, and 17% were 67 years or older. 53% of participants identified as female.

#### The Norwegian twin registry sample

The Norwegian Twin Registry comprises several population-based twin panels [42]. We used data from 1987 twins born between 1945 and 1960 who participated in a survey in 2016 (response rate: 64%). The data comprised responses from 528 monozygotic (MZ) female twins, 627 dizygotic (DZ) female twins, 375 MZ male twins, and 457 DZ male twins. In total, data were collected from 708 complete same-sexed twin pairs (i.e. 1416 individuals) and 571 single responders. Zygosity was determined by a questionnaire which has previously been shown to be highly accurate (> 97% correct classifications) [43]. The mean age was 63 years (*SD* = 4.5). 72% were aged 45 to 66 years and 28% were 67 years or older.

### Measures of well-being

We report the 37 items included in Study 1 (EFA) and Study 2 (CFA) in Table 1 (items in Study 3 are reported in the Supplementary Materials). Items originated from several well-established scales, including the Satisfaction with Life Scale [44], The Warwick–Edinburgh Mental Well-being Scale [45], The Mastery Scale [46], The Flourishing Scale [47], and international evaluations of well-being [48, 49].

**Table 1:** Well-being items included in the EFA and CFA

Item no	Question text	Scale or single item
Q1	In most ways my life is close to my ideal	SWLS^a^
Q2	The conditions of my life are excellent	SWLS^a^
Q3	I am satisfied with life	SWLS^a^
Q4	So far I have gotten the important things I want in life	SWLS^a^
Q5	If I could live my life over, I would change almost nothing	SWLS^a^
Q6	How often do you experience being interested in what you are doing?	ESS^b^
Q7	How often do you experience being absorbed in what you are doing?	ESS^b^
Q8	How often do you experience being enthusiastic about what you are doing?	ESS^b^
Q9	I’ve been feeling optimistic about the future	WEMWBS^c^
Q10	I’ve been feeling useful	WEMWBS^c^
Q11	I’ve been feeling relaxed	WEMWBS^c^
Q12	I’ve been dealing with problems well	WEMWBS^c^
Q13	I’ve been thinking clearly	WEMWBS^c^
Q14	I’ve been feeling close to other people	WEMWBS^c^
Q15	I’ve been able to make up my own mind about things	WEMWBS^c^
Q16	I have little control over what happens to me	Mastery scale^d^
Q17	Some of my problems I simply cannot solve	Mastery scale^d^
Q18	There is little I can do to change aspects of my life that are important	Mastery scale^d^
Q19	When faced with problems in my life I often feel helpless	Mastery scale^d^
Q20	Sometimes it feels like I am only pushed around in life	Mastery scale^d^
Q21	Overall, how satisfied are you with your life at the moment?	OECD^e,g^
Q22	Overall, to what extent do you experience what you're doing in life as worthwhile?	OECD^e,g^
Q23	In the last 7 days, to what extent have you been happy?	Adapted from OECD^e,g^
Q24	In the last 7 days, to what extent have you been worried?	Adapted from OECD^e,g^
Q25	In the last 7 days, to what extent have you been feeling down or sad?	Adapted from OECD^e,g^
Q26	My social relations are supportive and rewarding	Flourishing scale^f^
Q27	I actively contribute to the happiness and well-being of others	Flourishing scale^f^
Q28	Do you think your life is mostly full of experiences and rich, or mostly empty and boring?	Single item^g^
Q29	Overall, how happy with your life do you think you will be in 5 years?^h^	Single item^g^
Q30	In the last 7 days, to what extent have you been irritated?	Single item^g^
Q31	In the last 7 days, to what extent have you been invested/engaged?	Single item^g^
Q32	In the last 7 days, to what extent have you been calm and relaxed?	Single item^g^
Q33	In the last 7 days, to what extent have you been anxious?	Single item^g^
Q34	In the last 7 days, to what extent have you been stressed?	Single item^g^
Q35	How happy are you with your relationship with your children?	Single item^g^
Q36	How happy are you with your relationship with your friends?	Single item^g^
Q37	How happy are you with your relationship with your partner?	Single item^g^

^a^Satisfaction with Life Scale [44]

^b^European Social Survey (2013)

^c^The Warwick–Edinburgh Mental Well-being Scale [45]

^d^The Mastery Scale [46]

^e^OECD (2013)

^f^The Flourishing Scale [47]

^g^These items have been recommended for national monitoring of well-being in the Norwegian population (Nes et al., 2018)

^h^This item was not a part of the QoL 2020 survey and therefore only included in the EFA

### Data analysis

All analyses were conducted in the R Statistical Environment [50].

#### Study 1: exploratory factor analysis in the quality of life survey 2020

We conducted an EFA following a general approach outlined by Watkins [51]. Factor retention was based on three empirical criteria: Scree test, parallel analysis, and the minimum average partial (MAP) method. Scree tests plot eigenvalues from the correlation matrix to assess the location of any major drops in the graph [52]. Factors extracted after major drops are assumed to mostly represent error variance and are therefore not retained [51]. Parallel analysis compares observed and simulated eigenvalues (based on random data with an equal number of variables and sample size), retaining factors for which observed eigenvalues exceed simulated ones [53]. MAP separates common and unique variance in factor extraction: the lowest value is indicative of the point where all common variance is removed [51, 54]. The correlation matrix was estimated using Spearman correlation (MAP and parallel analysis were repeated using Pearson correlation to ensure robustness).

Squared multiple correlations were used in initial communality estimates. We used the weighted least squares solution for parallel analysis and factor extraction, considering the ordinal nature of the data, and the oblique promax factor rotation method to allow for intercorrelated factors. Factor extraction was repeated using Maximum Likelihood (ML) and ordinary least squares estimation and factor rotation using oblimin, to ensure the robustness of the factor structure. Missing data were treated with pairwise deletion.

We subjected the factor intercorrelation matrix to a new EFA, which can be done in hierarchical factor analysis [51], using the same empirical criteria. In addition, we examined the higher-order factor structure using the Schmid–Leiman transformation. EFA was conducted using the *psych* package [55].

#### Study 2: confirmatory factor analysis in the quality of life survey in Hallingdal 2019

Following the EFA, we examined the fit of the factor model identified in Study 1 in an independent sample. We used the diagonally weighted least squares estimator (DWLS), as this outperforms ML for ordinal data [56]. Missing data were treated with listwise deletion. The CFA suffered from some data loss (813 observations), as two relationship satisfaction items were asked a subset of the sample only (participants with a partner and/or children). We examined model fit both with and without these items.

Model fit was assessed using several fit indices, including the Comparative Fit Index (CFI), Tucker–Lewis Index (TLI), root mean square error of approximation (RMSEA), and Standardised Root Mean Square Residual (SRMR). Good model fit was determined by conventional thresholds [57]: CFI > 0.95, TLI > 0.95, RMSEA < .06, and SRMR < .08. The CFA was conducted using the *lavaan* [58] and *semPlot* [59] packages.

#### Study 3: examining genetic and environmental influences on well-being factors in the Norwegian twin registry (1945–1960 cohort)

In Study 3, we first conducted a CFA to test the fit of a hierarchical factor model with multiple first-order factors and a higher-order factor. This CFA used the DWLS estimator and model fit was assessed using similar fit indices as in Study 2. This analysis examined the fit of a model which was broadly similar to the model in Studies 1 and 2 in terms of including first-order factors and a higher-order factor, but the factors comprised partially different items. Optimism was included as a separate component, as it was measured by multiple items. Meaning in life was included as a distinct component, as the inclusion of this item in the “life satisfaction” component led to unreasonable parameter estimates with one communality estimate larger than 1.00 (i.e. a Heywood case). Three items measuring positive affect in daily life comprised a factor we called ‘positive affect’, as opposed to ‘positive activation’.

We examined genetic and environmental influences on the general well-being factors using biometric modelling [60, 61]. In this approach, phenotypic variation is explained by the influences of four components: additive genetic effects (A; correlated 1.0 for MZ twins and .5 for DZ twins), non-additive genetic effects (D; correlated 1.0 for MZ twins and .25 for DZ twins), shared environmental effects (C; correlated 1.0 for both MZ and DZ twins), and non-shared environmental effects (E; uncorrelated for both MZ and DZ twins).

Participants received an index score for each well-being factor based on the items which loaded on the given factor in the CFA in Study 3, if they had responded to more than half of the items in the index. Biometric analyses were conducted using mean scores on these indices as outcome variables. Individual item responses were standardised prior to computing index scores.

Correlational analyses were conducted to assess similarity in index scores across twins. Genetic and environmental influences on well-being components were examined using two multivariate models. The Cholesky model decomposes covariance between the latent A, C, and E variables and allows for estimating genetic and environmental correlations [62]. Multiple Cholesky models were estimated and compared for model fit, including models with A, D, and E effects (ADE); A, C, and E effects (ACE); A and E effects (AE); C and E effects (CE); and E effects only (E). The full ADCE model requires data from additional familial relationships and was therefore not estimated. Finally, we estimated a Common Pathway (CP) model, which assumes that covariation between index scores is explained by a latent well-being factor. The data were residualised on age and sex prior to conducting analyses. Biometric analyses were conducted using the *umx* [63] and *OpenMx* [64] packages.

## Results

### Study 1: exploratory factor analysis in the quality of life survey 2020

Initial analyses indicated that conducting EFA was appropriate. Most item correlations exceeded 0.30 and none exceeded 0.90 (see Supplementary Materials). Based on Bartlett’s [65] test of sphericity, the hypothesis that the correlation matrix was an identity matrix was rejected $({x}^{2}=414635.90,DF=666)$. The Kaiser–Meyer–Olkin [66] measure of sampling adequacy was acceptable. The overall value was $.97$ and values for the measured variables ranged from .94 to 99.

Empirical criteria suggested to retain from 6 (MAP) to 10 (parallel analysis) factors. Factor structures retaining from 6 to 10 factors were assessed for interpretability, meaningfulness, and symptoms of over- or underextraction. The most interpretable solution retained six factors. We called the first factor ‘life satisfaction’, as it comprised items assessing life satisfaction, experiencing life as meaningful, and optimism (Q21, Q22, Q28, Q29, Q1–Q5). The second factor, ‘positive activation’, comprised items assessing experiences of being engaged in and enthusiastic about one’s activities (Q6–Q8, Q31). Items loading on the third component, ‘autonomy’, queried about self-perceived (lack of) control over what happens in life, ability to find solutions, and feelings of hopelessness (Q16–Q20). The fourth factor, ‘well-functioning’, comprised several ‘functional’ aspects of well-being (e.g. cognition, problem-solving). It included items asking about recently having felt optimistic, been able to deal with problems well, been thinking clearly, and having felt close to other people (Q9–Q15). The fifth factor, ‘social’, included items assessing aspects of social relationships (Q26, Q27, Q35–Q37). Items loading on the final component, ‘absence of negative affect’, primarily assessed recently experienced negative affect (Q23–Q25, Q30, Q32–Q34). Standardised factor loadings are reported in Table 2 (empirical criteria and robustness analyses are reported in the Supplementary Materials).

**Table 2:** Factor loadings > .20 for six-factor solution with promax rotation (pattern matrix)

Item no	Description	LS	Standardised loadings					
			PA	AUT	WF	SOC	ANA	Communality^a^
Q1	Life close to ideal	**.99**						.75
Q3	Life satisfaction	**.96**						.79
Q4	Important things in life	**.87**						.56
Q2	Life conditions excellent	**.83**						.59
Q5	Change nothing	**.74**						.48
Q21	Overall life satisfaction	**.63**						.66
Q28	Life full of experiences and rich	**.49**	**.35**					.68
Q22	Life is worthwhile	**.47**	**.40**					.63
Q29	Happy with life in 5 years	**.39**	**.31**					.53
Q8	Enthusiastic		**1.01**					.77
Q7	Absorbed		**1.00**					.70
Q6	Interested		**.89**					.73
Q31	Last 7 days, invested/engaged		**.56**		.22			.52
Q18	Unable to change aspects of life			**.85**				.57
Q17	Cannot solve problems			**.79**				.51
Q16	Little control over what happens			**.65**				.42
Q19	Helpless when faced with problems			**.64**				.60
Q20	Pushed around in life			**.43**				.49
Q13	Thinking clearly				**.89**			.62
Q12	Dealing with problems well				**.73**			.61
Q15	Able to make up my own mind				**.63**			.39
Q14	Feeling close to other people				**.46**	**.37**		.50
Q11	Feeling relaxed				**.42**		**.35**	.52
Q10	Feeling useful		.26		**.42**			.53
Q9	Feeling optimistic about the future	.26			**.26**			.48
Q26	Supportive and rewarding relations					**.77**		.60
Q36	Happy with relationship with friends					**.66**		.47
Q27	Contribute to happiness of others					**.64**		.51
Q35	Happy with relationship with children					**.63**		.33
Q37	Happy with relationship with partner					**.62**		.40
Q24	Last 7 days, worried						**.86**	.62
Q33	Last 7 days, anxious						**.84**	.63
Q34	Last 7 days, stressed						**.80**	.55
Q25	Last 7 days, down or sad						**.73**	.67
Q30	Last 7 days, irritated						**.56**	.37
Q32	Last 7 days, calm and relaxed				.24		**.43**	.51
Q23	Last 7 days, happy	.20	.21			.21	**.27**	.61

^a^Communality is the proportion of variance explained by the factors [51]. LS represents ‘life satisfaction’; PA represents ‘positive activation’; AUT represents ‘autonomy’; WF represents ‘well-functioning’; SOC represents ‘social’; and ANA represents ‘absence of negative affect’. Loadings > .20 are displayed and loadings > .30 are in bold. Item descriptions are based on the full items, as reported in Table 1. The loadings from the pattern matrix reflect regression-like coefficients and may exceed ± 1 [51]. The EFA was conducted in the QoL 2020 sample

Several variables had complex cross-loadings. Q22, Q28, and Q29 loaded on both the life satisfaction and positive activation factors; Q9 loaded on both the well-functioning and life satisfaction factors; Q10 loaded on both the well-functioning and positive activation factors; Q11 loaded on both the well-functioning and absence of negative affect factors; Q14 loaded on both the well-functioning and the social factors; Q23 loaded on the absence of negative affect, positive activation, social, and life satisfaction factors; Q31 loaded on both the positive activation and well-functioning factors; and Q32 loaded on both the absence of negative affect and well-functioning factors.

All criteria suggested that one higher-order factor could be extracted. We called this ‘the general happiness factor’ (the ‘h-factor’). With one exception, all first-order factors had loadings > .70 on this higher-order factor. The standardised loadings to the higher-order factor were .89 for life satisfaction, .82 for positive activation, .67 for autonomy, .83 for well-functioning, .75 for social, and .72 for absence of negative affect.

### Study 2: confirmatory factor analysis in the quality of life survey in Hallingdal 2019

**Fig. 1:**
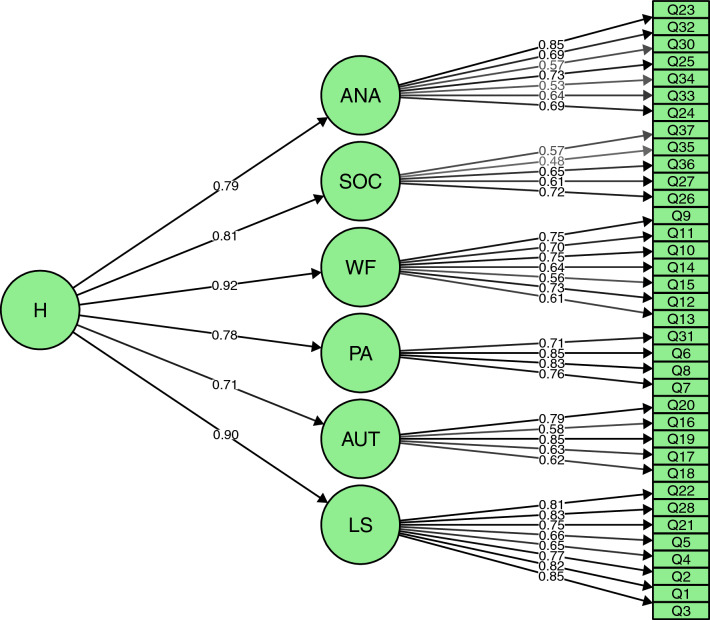
CFA Results of Model with Six First-Order Factors and One Higher-Order Factor. *ANA* absence of negative affect; *SOC* social; *WF* well-functioning; *PA* positive activation; *AUT* autonomy; and *LS* life satisfaction. The plot depicts the standardised factor loadings. The CFA was conducted in the QoL 2019 sample

In Study 2, we examined the fit of the factor model identified in Study 1 in an independent sample. The factor structure was pre-defined to be identical with the structure identified in Study 1: individual items loaded on one of six well-being factors, which all loaded on a single higher-order factor. All statistics indicated good model fit: ${\chi }^{2}=1528.732 \left(df=588, p< .001\right), \mathrm{RMSEA}=.035 \left(90\mathrm{\% CI}:.033, .037\right),\mathrm{ SRMR}=.053,\mathrm{ CFI}=.987$ and $\mathrm{TLI}=.986.$ (see Fig. 1).

### Study 3: biometric modelling in the Norwegian twin registry sample

All model fit statistics indicated that the model with a higher-order and multiple first-order factors was a good fit to the data: ${\chi }^{2}=1313.827 \left(df=489, p< .001\right), \mathrm{ RMSEA}=.035 \left(90\mathrm{\% CI}:.033, .038\right),\mathrm{ SRMR}=.053,\mathrm{ CFI}=.976$ and $\mathrm{TLI}=.974$.

**Fig. 2:**
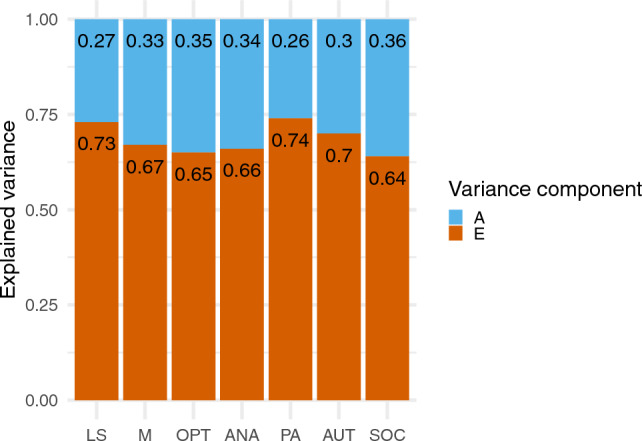
Estimated Genetic and Environmental Effects on Well-being Components. ‘A’ represents additive genetic effects and ‘E’ represents non-shared environmental effects. ‘LS’ represents life satisfaction; ‘M’ represents meaning in life; ‘OPT’ represents optimism; ‘ANA’ represents absence of negative affect, ‘PA’ represents positive affect; ‘AUT’ represents autonomy; ‘SOC’ represents Social. We report confidence intervals for the A and E variance components in the Supplementary Materials

Index score correlations were systematically higher for MZ than DZ co-twins, indicative of genetic influence on all well-being factors (see Table 3). The AE Cholesky model had best fit to the data, indicated both by AIC and RMSEA values (see Table 4). Moderate genetic influence and substantial non-shared environmental influence was observed for all first-order well-being factors, with heritability estimates ranging from .26 to .36 (see Fig. 2; parameter estimates, confidence intervals, and genetic and environmental correlations are reported in the Supplementary Materials).

**Table 3:** Twin correlations for index scores

Index mean score	Monozygotic		Dizygotic	
	Twin 1	Twin 2	Twin 1	Twin 2
Life Satisfaction twin 1	1.00	.252	1.00	.146
Meaning twin 1	1.00	.283	1.00	.150
Optimism twin 1	1.00	.337	1.00	.233
Absence of negative affect twin 1	1.00	.382	1.00	.132
Positive affect twin 1	1.00	.242	1.00	.126
Autonomy twin 1	1.00	.336	1.00	.081
Social twin 1	1.00	.387	1.00	.104

**Table 4:** Fit statistics for multivariate twin models

Model	df	Δ Fit	Δ df	p	AIC	Δ AIC	RMSEA [95% CI]
Multivariate cholesky (ACE)	91				27,870.19	–	.020 [.013, .026]
Multivariate cholesky (ADE)	91	− 12.512	0		27,857.68	− 12.512	.018 [.011, .025]
**Multivariate cholesky (AE)**	**63**	**3.951**	**28**	**1.000**	**27,818.14**	− **52.049**	.015 [.007, .021]
Multivariate cholesky (CE)	63	49.760	28	.007	27,863.95	− 6.240	.021 [.015, .026]
Multivariate cholesky (E)	35	230.124	56	< .001	27,988.31	118.124	.031 [.026, .036]

The model fit statistics for the best-fitting model indicated by RMSEA and AIC values are in bold

**Fig. 3:**
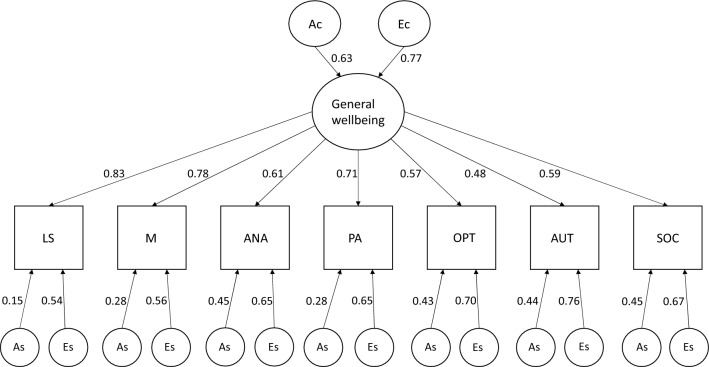
Parameter Estimates from Common Pathway Model. We note that this model had worse fit compared with an AE Cholesky model but good fit indicated by RMSEA

The AE CP model had worse fit compared with the AE Cholesky model ($AIC=28001.840; RMSEA= .032, 95\% CI [.027, .036] )$. The heritability of the latent well-being factor estimated in the AE CP model was 40% (see Fig. 3).

## Discussion

Across more than 21,500 participants, we identified a well-being structure comprising six first-order factors and one higher-order factor. This model had excellent fit in an independent sample. All well-being factors, including the higher-order happiness factor, showed moderate genetic and substantial non-shared environmental influence.

Our results suggest that the structure of well-being encompasses both hedonic and eudaimonic aspects, which were subsumed in broader factors. The factor model included both well-being facets conceptualised as ‘hedonic’ [7], like the presence of positive and absence of negative affect, and aspects classified as ‘eudaimonic’ [9], such as well-functioning. In addition, social aspects of well-being, emphasised in recent models [11], comprised a first-order factor with a strong loading on the higher-order factor. One previous study found that a best-fitting hierarchical model comprised hedonic, eudaimonic, and social higher-order factors [4].

Our finding of six first-order well-being components is in partial agreement with previous studies which have also identified multiple general well-being factors [4, 15–17, 19–22, 30, 67–72]. Specific well-being components identified across studies are likely to vary, in part because well-being may be measured using different items and scales [69], which should be kept in mind when interpreting our findings. Furthermore, some items loaded on more than one factor in our EFA. We note, however, that global fit statistics from the subsequent CFA indicated good model fit.

All first-order factors loaded strongly on a higher-order well-being factor (the h-factor). This corroborates findings from several studies which have found evidence for a general higher-order factor in hierarchical or bifactor models of well-being [4, 19–22, 26, 27, 30, 67]. The hierarchical model can be interpreted as nested within the bifactor model [51, 73, 74]. Thus, our findings support converging evidence, from studies applying both hierarchical and bifactor models, in identifying one general well-being factor. We note that random measurement error is typically contained at the item level and not present in the higher-order latent factor.

We note that these well-being factors refer to statistical constructs. Theoretical work is needed to better understand what the higher-order well-being factor reflects. One possibility is that it broadly corresponds with ‘overall perceived enjoyment and fulfilment with life’, as proposed by Disabato et al. [18]. A similar higher-order factor has also been theorised to represent a ‘positive orientation’ towards life [40, 75], with one study indicating that positive orientation may reflect a common factor for hedonic and eudaimonic well-being [76]. However, interpreting the general factor is difficult given the multidimensional nature of well-being, and some have noted that the single factor may not actually reflect a positive construct [23].

Our study yields novel findings regarding the genetic and environmental architecture of well-being. All first-order well-being factors showed moderate genetic and substantial non-shared environmental influence. Several heritability estimates are close to previously reported estimates, e.g. we estimate the heritability of life satisfaction to be 27%, compared with 32% in one previous meta-analysis [36]. Heterogeneity in well-being measures likely contributes to variation heritability estimates across studies [36], together with other factors, such as measurement error. Age differences could also be a contributing factor to varying heritability estimates across studies and samples. However, Bartels [36] did not find a substantial effect of age on heritability estimates.

The higher-order factor had a heritability estimated to 40% (in the Common Pathway model), which is close to what has been reported for well-being (36%) and somewhat higher than for life satisfaction (32%) [36]. This estimate is lower than what has been reported for a latent ‘Well-being’ factor (48%) [20] and latent ‘mental well-being’ factor (72%) [38].

### Strengths and limitations

Our study has several strengths. We used three large and independent samples to examine the structure of well-being, two of which were population based. Well-being was measured using multiple items from several questionnaires with different well-being conceptualisations. Thirdly, we used both EFA and CFA to examine the factor structure of well-being and its replicability, leveraging both exploratory and confirmatory factor analytic approaches.

Our study also has several limitations. First, although well-being components were broadly corresponding across studies, the factor structure was modelled with minor differences in the twin sample due to partially different items. However, this model also had good fit to the data, providing further support for a hierarchical well-being model. Second, data were residualised on sex but possible sex differences in genetic and environmental effects were not investigated. Findings have been inconclusive with regards to sex differences in these effects on well-being [36]. Third, our samples consisted only of Norwegian adults. Aspects of well-being which are emphasised vary across cultures [77], leaving the generalisability of the identified well-being structure in our study unclear. Fourth, a theoretical framework for explaining the structure of well-being we identify is lacking. There have been calls for more emphasis on theoretical work alongside factor analysis [78] and in well-being research [79]. Fifth, previous studies have tested the external validity of bifactor models for the p-factor [80]. Our study is limited in that it does not evaluate the external validity of the factor models. Sixth, data collection for QoL 2020 was conducted during the first national lockdown in Norway related to the Coronavirus Disease 2019 pandemic, possibly influencing responses. We note that model identified using EFA had excellent fit in the QoL 2019 survey data, collected before the pandemic outbreak.

### Implications

Our findings have implications for understanding the structure of well-being. Firstly, hedonic and eudaimonic well-being were not distinguishable as distinct components but included in broader factors. Thus, models which conceptualise these as separate components may not accurately capture the structure of well-being. Secondly, we identified a higher-order happiness factor, which underlies the structure of well-being. Thirdly, genetic effects on well-being factors, including the higher-order factor, were moderate, with the majority of variance explained by non-shared environmental factors.

Our findings may have multiple implications for future research. Examining the content of the higher-order happiness factor, its correlates, and the structure of genetic and environmental influences on this factor could be a useful aim for future studies. Furthermore, examining general well-being factors in non-Scandinavian cultures is desirable to better understand generalisability and cultural influences on well-being. Future research efforts could use longitudinal data to investigate stability and change in general well-being factors. One previous study found a high degree of stability in a latent well-being factor across six years [71].

## Conclusion

We conducted three studies to advance knowledge on the structure of well-being and its genetic and environmental architecture. We identified six first-order well-being factors which all loaded on a higher-order well-being factor. The model had excellent fit in an independent sample. All well-being components were moderately influenced by genes and substantially influenced by non-shared environmental factors. Our findings have implications for understanding the structure of well-being, theories of well-being, and future research efforts.

## Supplementary Information

Below is the link to the electronic supplementary material.

Supplementary file1 (DOCX 485 KB)
