# Supplementary material for: The structure of well-being: a single underlying factor with genetic and environmental influences
Source: Qual Life Res. 2023 May 20;32(10):2805–16. doi: 10.1007/s11136-023-03437-7 (PMC10199429; doi:10.1007/s11136-023-03437-7)
Supplement: Supplementary file 1 — Supplementary file1 (DOCX 485 KB) [file 11136_2023_3437_MOESM1_ESM.docx]

**Supplementary materials for manuscript entitled “The structure of wellbeing–a single underlying factor with genetic and environmental influences”**

^1^Corresponding author: Ludvig Daae Bjørndal

Department of Psychology, University of Oslo, PO Box 1094, 0317, Oslo, Norway.

E-mail: [l.d.bjorndal@psykologi.uio.no](mailto:l.d.bjorndal@psykologi.uio.no)

**Table of Contents**

- Details and characteristics of the samples
  - Quality of Life Survey 2020
  - Quality of Life Survey in Hallingdal 2019
  - The Norwegian Twin Registry
- Wellbeing items and number of responses in each sample
  - Quality of Life Survey 2020
  - Quality of Life Survey in Hallingdal 2019
  - The Norwegian Twin Registry
- Study 1: EFA
  - Correlation matrix for wellbeing items.
  - Criteria for retaining factors
  - Factor structures from 10 to 7 factors
  - Criteria for retaining factors in hierarchical EFA
  - Factor extraction using Pearson correlation
  - Factor extraction with ML
  - Factor extraction with OLS
  - Factor extraction with principal axis
  - Factor extraction with principal axis and oblimin
  - Schmid-Leiman transformation
- Study 2: CFA
  - CFA without social items.
- Study 3: Genetically informative analyses
  - Correlational analyses
  - Parameter estimates, confidence intervals, and genetic and environmental correlations from multivariate Cholesky AE model
- Code
  - Study 1
  - Study 2
  - Study 3
- References

**Details and characteristics of the samples**

***Quality of Life Survey 2020***

The Quality of Life Survey 2020 (QoL 2020) was conducted by Statistics Norway in March 2020. The sample is population-based and participants came from all regions of Norway. The full sample size was 17,417. Table S1 reports the number of responses for each item. More details can be found on the website of Statistics Norway: <https://www.ssb.no/sosiale-forhold-og-kriminalitet/artikler-og-publikasjoner/livskvalitetsundersokelsen-2020>

***Quality of Life Survey in Hallingdal 2019***

The Quality of Life Survey in Hallingdal 2019 (QoL 2019) was conducted in March and April 2019. The full sample size was 2125. Table S3 reports the number of responses for each item. More information can be found on the website of Statistics Norway: <https://www.ssb.no/sosiale-forhold-og-kriminalitet/artikler-og-publikasjoner/pilotundersokelse-om-livskvalitet-i-hallingdal>

In the Quality of Life Survey 2020 and Quality of Live Survey in Hallingdal 2019, the response alternatives also included ‘Don’t know’ and ‘Don’t wish to answer’. As these response alternatives are not part of the wellbeing scales themselves, participants who chose these response alternatives were excluded from subsequent analyses. We report the number of respondents choosing one of these two options in Table S1 and S3.

***The Norwegian Twin Registry***

The twin sample was recruited from a population-based sample in the Norwegian Twin Registry (NTR). The full sample size was 1987. Table S6 reports the number of responses for each item. NTR consists of a number of different cohorts. NTR has been described elsewhere (Nilsen et al., 2016).

**Wellbeing items and number of responses in each sample.**

**Table S1.**

*Number of Responses for Each Item in the QoL 2020 Study (N = 17,417)*.

| **Item code** | **Number of responses** | **Number of “Don’t know” or “Do not wish to answer” responses** |
| --- | --- | --- |
| Q21 | 17,410 | 7 |
| Q22 | 17,381 | 36 |
| Q28 | 17,398 | 19 |
| Q29 | 17,209 | 208 |
| Q1 | 17,411 | 6 |
| Q2 | 17,408 | 9 |
| Q3 | 17,411 | 6 |
| Q4 | 17,409 | 8 |
| Q5 | 17,404 | 13 |
| Q6 | 17,407 | 10 |
| Q7 | 17,407 | 10 |
| Q8 | 17,405 | 12 |
| Q16 | 17,406 | 11 |
| Q17 | 17,406 | 11 |
| Q18 | 17,401 | 16 |
| Q19 | 17,407 | 10 |
| Q20 | 17,409 | 8 |
| Q9 | 17,404 | 13 |
| Q10 | 17,411 | 6 |
| Q11 | 17,411 | 6 |
| Q12 | 17,412 | 5 |
| Q13 | 17,409 | 8 |
| Q14 | 17,413 | 4 |
| Q15 | 17,412 | 5 |
| Q26 | 17,408 | 9 |
| Q27 | 17,405 | 12 |
| Q23 | 17,413 | 4 |
| Q24 | 17,414 | 3 |
| Q25 | 17,414 | 3 |
| Q30 | 17,411 | 6 |
| Q31 | 17,415 | 2 |
| Q32 | 17,411 | 6 |
| Q33 | 17,412 | 5 |
| Q34 | 17,413 | 4 |
| Q35 | 12,361 | 10 |
| Q36 | 17,393 | 24 |
| Q37 | 13,367 | 14 |

*Notes*. Q35 (satisfaction with relationship to children) and Q37 (satisfaction with relationship to partner) were only showed to participants who had indicated that they had children and/or a partner, respectively.

**Table S2.**

*Response Formats for Items in Study 1 and 2 Samples*

| **Item codes** | **Response format** |
| --- | --- |
| Q21 | Not happy (0) - Very happy (10) |
| Q22 | Not worthwhile at all (0) - Very worthwhile (10) |
| Q28 | Always empty and boring (0) – Always rich and giving (10) |
| Q29 | Not happy at all (0) - Very happy (10) |
| Q1-Q5 | Totally disagree (1) - Totally agree (7) |
| Q6-Q8 | Never (0) - All the time (10) |
| Q16-Q20 | Totally disagree (1) - Totally agree (5) |
| Q9-Q15 | Not at all (1) - All the time (5) |
| Q23-Q25, Q30-Q34 | Not at all (0) - To a large degree (10) |
| Q26, Q27 | Totally disagree (0) - Totally agree (10) |
| Q35-Q37 | Not happy at all (0) - Very happy (10) |

*Notes*. Some items were recoded so that all items reflected higher wellbeing (i.e., these items were reversed). Recoded items were: Q16-Q20, Q24, Q25, Q30, Q33, Q34.

***Items in the Quality of Life Survey in Hallingdal 2019 study***

The majority of the items in the QoL 2019 study were similar to the QoL 2020 study. One item was omitted in the QoL 2019 data: Q29. The QoL 2019 study also included some additional items which assessed negative affect, but we did not include these in the CFA analyses, as these were not included in the dataset used for the EFA.

**Table S3.**

*Number of Responses to Each Item in the QoL 2019 Study (N = 2125)*.

| **Item code** | | **Number of responses** | **Number of “Don’t know” or “Do not wish to answer” responses** | | |
| --- | --- | --- | --- | --- | --- |
| Q21 | | 2125 | | | 0 |
| Q22 | | 2120 | | | 5 |
| Q28 | | 2124 | | | 1 |
| Q1 | | 2125 | | | 0 |
| Q2 | | 2123 | | | 2 |
| Q3 | | 2124 | | | 1 |
| Q4 | | 2124 | | | 1 |
| Q5 | | 2123 | | | 2 |
| Q6 | | 2125 | | | 0 |
| Q7 | | 2123 | | | 2 |
| Q8 | | 2122 | | | 3 |
| Q16 | | 2125 | | | 0 |
| Q17 | | 2124 | | | 1 |
| Q18 | | 2125 | | | 0 |
| Q19 | | 2125 | | | 0 |
| Q20 | | 2125 | | | 0 |
| Q9 | | 2124 | | | 1 |
| Q10 | | 2125 | | | 0 |
| Q11 | | 2125 | | | 0 |
| Q12 | | 2125 | | | 0 |
| Q13 | | 2125 | | | 0 |
| Q14 | | 2125 | | | 0 |
| Q15 | | 2124 | | | 1 |
| Q26 | | 2122 | | | 3 |
| Q27 | | 2121 | | | 4 |
| Q23 | | 2125 | | | 0 |
| Q24 | | 2125 | | | 0 |
| Q25 | | 2125 | | | 0 |
| Q30 | | 2124 | | | 1 |
| Q31 | | 2125 | | | 0 |
| Q32 | | 2123 | | | 2 |
| Q33 | | 2125 | | | 0 |
| Q34 | | 2125 | | | 0 |
| Q35 | | 1542 | | | 1 |
| Q36 | | 2124 | | | 1 |
| Q37 | | 1616 | | | 3 |

*Notes*. Q35 (satisfaction with relationship to children) and Q37 (satisfaction with relationship to partner) were only showed to participants who had indicated that they had children and/or a partner, respectively.

**Table S4.**

*Items in the Norwegian Twin Registry 2016 Sample*

| **Item code** | **Question** |
| --- | --- |
| S_22_1 | In most ways my life is close to my ideal. |
| S_22_2 | The conditions of my life are excellent. |
| S_22_3 | I am satisfied with my life. |
| S_22_4 | So far I have gotten the important things I want in life. |
| S_22_5 | If I could live my life over, I would change almost nothing. |
| S_23 | Overall, would you say that what you are doing in life is worthwhile? |
| S_24_1 | Have you been bothered by any of the following during the last two weeks: being afraid or anxious. |
| S_24_2 | Have you been bothered by any of the following during the last two weeks: Nervousness or shakiness inside. |
| S_24_3 | Have you been bothered by any of the following during the last two weeks: Feeling hopeless about the future. |
| S_24_4 | Have you been bothered by any of the following during the last two weeks: Feeling blue. |
| S_24_5 | Have you been bothered by any of the following during the last two weeks: Worrying too much about things. |
| S_24_6 | Have you been bothered by any of the following during the last two weeks: Feeling like everything is an effort. |
| S_24_7 | Have you been bothered by any of the following during the last two weeks: Felt tense or keyed up. |
| S_24_8 | Have you been bothered by any of the following during the last two weeks: Suddenly scared for no reason. |
| S_25_1_1 | How often do you experience the following in your everyday life: Feel glad about something. |
| S_25_1_2 | How often do you experience the following in your everyday life: Feel happy. |
| S_25_1_3 | How often do you experience the following in your everyday life: Feel joyful, like everything is going your way. |
| S_26_1 | I can always manage to solve difficult problems if I try hard enough. |
| S_26_2 | If I am in trouble, I can usually think of a solution. |
| S_26_3 | If someone opposes me, I can find the means and ways to get what I want. |
| S_26_4 | I am confident that I could deal efficiently with unexpected events. |
| S_26_5 | I can remain calm when facing difficulties because I can rely on my coping abilities. |
| S_31_1 | In uncertain times, I usually expect the best. |
| S_31_2 | If something can go wrong for me, it will. |
| S_31_3 | I am always optimistic about my future. |
| S_31_4 | I hardly ever expect things to go my way. |
| S_31_5 | I rarely count on good things happening to me. |
| S_31_6 | Overall, I expect more good things to happen to me than bad. |
| S_32_1 | I am very happy with our relationship. |
| S_32_2 | My partner and I have problems in our relationship. |
| S_32_3 | My partner is generally understanding. |
| S_32_4 | I am satisfied with my relationship with my partner. |
| S_32_5 | We agree on how children should be raised. |

**Table S5.**

*Scales and Response Formats for Items in the Study 3 Sample.*

| **Item codes** | **Scale** | **Response format** | |
| --- | --- | --- | --- |
| S_22_1, S_22_2, S_22_3, S_22_4, S_22_5 | SWLS^1^ | | Totally disagree (1) - Totally agree (7) |
| S_23 | OECD^2^ | | Not meaningful at all (0) - Very meaningful (10) |
| S_31_1, S_31_2, S_31_3, S_31_4, S_31_5, S_31_6 | LOT-R^3^ | | Totally disagree (1) – Totally agree (5) |
| S_24_1, S_24_2, S_24_3, S_24_4, S_24_5, S_24_6, S_24_7, S_24_8 | SCL-8^4^ | | Not bothered (1) – Very much bothered (4) |
| S_25_1_1, S_25_1_2, S_25_1_3 | DES^5^ | | Rarely/Never (1) – Very often (5) |
| S_26_1, S_26_2, S_26_3, S_26_4, S_26_5 | GSE^6^ | | Not correct (1) – Totally correct (4) |
| S_32_1, S_32_2, S_32_3, S_32_4, S_32_5 | RSS^7^ | | Strongly disagree (1) – Strongly agree (6) |

*Notes*. ^1^Satisfaction with Life Scale (Diener et al., 1985), ^2^OECD (2013), ^3^Revised Life Orientation Test (Scheier et al., 1994), ^4^Symptom Checklist-8 (Tambs & Røysamb, 2014), ^5^Differential Emotion Scale (Izard et al., 1993), ^6^General Self-Efficacy scale (Leganger et al., 2000; Tambs & Røysamb, 2014), ^7^Relationship Satisfaction Scale (Røysamb et al., 2014). Some items were recoded so that all items reflected higher wellbeing (i.e., these items were reversed). Recoded items were: S_24_1, S_24_2, S_24_3, S_24_4, S_24_5, S_24_6, S_24_7, S_24_8, S_31_2, S_31_4, S_31_5, S_32_2.

**Table S6.**

*Number of Responses in the Norwegian Twin Registry Sample (N = 1987).*

| **Item code** | | **Number of responses** | |
| --- | --- | --- | --- |
| S_22_1 | | 1945 | |
| S_22_2 | | 1947 | |
| S_22_3 | | 1969 | |
| S_22_4 | | 1950 | |
| S_22_5 | | 1964 | |
| S_23 | | 1956 | |
| S_24_1 | | 1890 | |
| S_24_2 | | 1908 | |
| S_24_3 | | 1903 | |
| S_24_4 | | 1907 | |
| S_24_5 | | 1915 | |
| S_24_6 | | 1908 | |
| S_24_7 | | 1907 | |
| S_24_8 | | 1899 | |
| S_25_1_1 | | 1976 | |
| S_25_1_2 | | 1969 | |
| S_25_1_3 | | 1966 | |
| S_26_1 | | 1957 | |
| S_26_2 | | 1954 | |
| S_26_3 | | 1936 | |
| S_26_4 | | 1941 | |
| S_26_5 | | 1954 | |
| S_31_1 | | 1961 | |
| S_31_2 | | 1952 | |
| S_31_3 | | 1961 | |
| S_31_4 | | 1949 | |
| S_31_5 | | 1963 | |
| S_31_6 | | 1968 | |
| S_32_1 | | 1622 | |
| S_32_2 | | 1604 | |
| S_32_3 | | 1614 | |
| S_32_4 | | 1622 | |
| S_32_5 | | 1576 | |

**Study 1: EFA conducted in the QoL 2020 sample.**

**Figure S1.**

*Correlation Matrix of Wellbeing Items.*

***Criteria for retaining factors in initial EFA***

**Figure S2.**

*Scree Plot*.

**Figure S3.**

*Parallel Analysis.*

**Table S7.**

*MAP*.

| **Number of factors** | **MAP** |
| --- | --- |
| 1 | .0178 |
| 2 | .0136 |
| 3 | .0121 |
| 4 | .0121 |
| 5 | .0107 |
| 6 | .0098 |
| 7 | .0099 |
| 8 | .0106 |
| 9 | .0116 |
| 10 | .0125 |

The Velicer MAP achieved a minimum of .01 with 6 factors.

***Factor structures***

**Figure S4.**

**Figure S5.**

**Figure S6.**

**Figure S7.**

***Criteria for retaining factors in hierarchical EFA***

**Figure S8.**

*Scree Plot.*

**Figure S9.**

*Parallel Analysis.*

**Table S8.**

*MAP.*

| **Number of factors** | **MAP** |
| --- | --- |
| 1 | .058 |
| 2 | .126 |
| 3 | .225 |

The Velicer MAP achieved a minimum of .06 with 1 factor.

***Factor extraction using Pearson correlation***

**Figure S10.**

*Correlation Matrix of Wellbeing Items Estimated With Pearson Correlation.*

******

**Figure S11.**

******

***Factor extraction with ML***

**Figure S12.**

******

***Factor extraction with OLS***

**Figure S13.**

******

***Factor extraction with principal axis***

**Figure S14.**

******

***Factor rotation with principal axis estimation and oblimin***

**Figure S15.**

******

***Schmid-Leiman Transformation***

Higher-order EFA can also be conducted using the Schmid-Leiman (S-L) transformation, which modifies the higher-order model into orthogonal sources of variance, facilitating interpretation (Watkins, 2020).

**Table S9.**

*Schmid-Leiman Factor Loadings > 0.2.*

| **Item** | **g** | **F1** | **F2** | **F3** | **F4** | **F5** | **F6** | **h2** | **u2** | **p2** |
| --- | --- | --- | --- | --- | --- | --- | --- | --- | --- | --- |
| Q21 | .74 | .29 |  |  |  |  |  | .66 | .34 | .83 |
| Q22 | .72 | .21 |  | .23 |  |  |  | .63 | .37 | .83 |
| Q28 | .76 | .22 |  | .20 |  |  |  | .68 | .32 | .84 |
| Q29 | .64 |  |  |  |  |  |  | .53 | .47 | .77 |
| Q1 | .74 | .45 |  |  |  |  |  | .75 | .25 | .73 |
| Q2 | .65 | .38 |  |  |  |  |  | .59 | .41 | .71 |
| Q3 | .77 | .44 |  |  |  |  |  | .79 | .21 | .75 |
| Q4 | .63 | .40 |  |  |  |  |  | .56 | .44 | .70 |
| Q5 | .61 | .34 |  |  |  |  |  | .48 | .52 | .77 |
| Q6 | .68 |  |  | .51 |  |  |  | .73 | .27 | .64 |
| Q7 | .61 |  |  | .58 |  |  |  | .70 | .30 | .53 |
| Q8 | .66 |  |  | .58 |  |  |  | .77 | .23 | .56 |
| Q16 | .43 |  |  |  |  | .48 |  | .42 | .58 | .44 |
| Q17 | .40 |  |  |  |  | .59 |  | .51 | .49 | .31 |
| Q18 | .43 |  |  |  |  | .63 |  | .57 | .43 | .32 |
| Q19 | .59 |  |  |  |  | .47 |  | .60 | .40 | .59 |
| Q20 | .60 |  |  |  |  | .32 |  | .49 | .51 | .73 |
| Q9 | .66 |  |  |  |  |  |  | .48 | .52 | .90 |
| Q10 | .67 |  |  |  |  |  | .24 | .53 | .47 | .85 |
| Q11 | .62 |  | .25 |  |  |  | .24 | .52 | .48 | .73 |
| Q12 | .66 |  |  |  |  |  | .41 | .61 | .39 | .71 |
| Q13 | .58 |  |  |  |  |  | .50 | .62 | .38 | .55 |
| Q14 | .60 |  |  |  | .25 |  | .26 | .50 | .50 | .72 |
| Q15 | .49 |  |  |  |  |  | .36 | .39 | .61 | .63 |
| Q26 | .58 |  |  |  | .51 |  |  | .60 | .40 | .56 |
| Q27 | .53 |  |  |  | .43 |  |  | .51 | .49 | .55 |
| Q23 | .73 |  |  |  |  |  |  | .61 | .39 | .88 |
| Q24 | .51 |  | .60 |  |  |  |  | .62 | .38 | .43 |
| Q25 | .65 |  | .51 |  |  |  |  | .67 | .33 | .62 |
| Q30 | .46 |  | .39 |  |  |  |  | .37 | .63 | .57 |
| Q31 | .63 |  |  | .32 |  |  |  | .52 | .48 | .76 |
| Q32 | .62 |  | .30 |  |  |  |  | .51 | .49 | .76 |
| Q33 | .53 |  | .58 |  |  |  |  | .63 | .37 | .45 |
| Q34 | .46 |  | .56 |  |  |  |  | .55 | .45 | .38 |
| Q35 | .39 |  |  |  | .42 |  |  | .33 | .67 | .46 |
| Q36 | .53 |  |  |  | .44 |  |  | .47 | .53 | .59 |
| Q37 | .44 |  |  |  | .42 |  |  | .40 | .60 | .50 |

*Notes*. ‘p2’ here represents the percent of the common variance for each variable, which is general factor variance. The eigenvalues were 13.48 for *g*, 1.09 for F1, 1.71 for F2, 1.25 for F3, 1.14 for F4, 1.36 for F5 and .84 for F6.

On average, the general factor explained 64% of the common variance in the measured variables. Therefore, The Schmid-Leiman transformation also supports the extraction of a higher-order factor.

**Study 2: Confirmatory Factor Analysis Conducted in the QoL 2019 Sample.**

Due some missing data in the Confirmatory Factor Analysis (CFA) reported in the manuscript, we also examined the fit of a 6-factor model with a higher-order happiness factor, excluding the two social items which explained the substantial loss of data. The number of observations used in these analyses was 2103. We report the results of these analyses in Table S10. The fit was comparable to that of the model including the two social items, as reported in the manuscript.

**Table S10.**

*Model-Fit Statistics From CFA Excluding Social Items Related to Partners and Children.*

| **Model** | $\chi^{\boldsymbol{2}}$ | **df** | **p** | **CFI** | **TLI** | **SRMR** | **RMSEA [90% CI]** |
| --- | --- | --- | --- | --- | --- | --- | --- |
| 6 factor model with a higher-order factor | 2267.255 | 521 | .000 | .986 | .984 | .053 | .040 [.038, .042] |

**Figure S16.**

*CFA Loadings Excluding the Two Social Items With Low N.*

**Study 3: Genetically Informative Analyses in the Norwegian Twin Registry Sample.**

***Correlational analyses***

The index scores were calculated so that a participant only received a mean of the items he or she had answered, if the participant had answered at least 50% of the items in each index. The means were calculated based on the standardised items.

Before conducting univariate biometric analyses, we examined correlations between Twin 1 and Twin 2 for monozygotic (MZ) and dizygotic (DZ) twins for all standardised mean scores. All of these analyses yielded higher correlations for MZ twins compared with DZ twins, which gives a crude indication of genetic influence on the mean scores. These correlations are reported in the main manuscript (Table 3).

***Multivariate Cholesky AE model***

Table S11 reports the standardized parameter estimates from the multivariate AE Cholesky model. Table S12 reports the estimated genetic and environmental correlations.

**Table S11.**

*Parameter Estimates from AE Cholesky model*.

| **Index** | **A1** | **A2** | **A3** | **A4** | **A5** | **A6** | **A7** | **E1** | **E2** | **E3** | **E4** | **E5** | **E6** | **E7** |
| --- | --- | --- | --- | --- | --- | --- | --- | --- | --- | --- | --- | --- | --- | --- |
| LS | .516 |  |  |  |  |  |  | .857 |  |  |  |  |  |  |
| M | .475 | .318 |  |  |  |  |  | .492 | .657 |  |  |  |  |  |
| OPT | .405 | -.021 | .429 |  |  |  |  | .248 | .176 | .748 |  |  |  |  |
| ANA | .337 | .073 | .158 | .442 |  |  |  | .421 | .117 | .067 | .682 |  |  |  |
| PA | .387 | .168 | .163 | -.106 | .222 |  |  | .442 | .156 | .140 | .052 | .702 |  |  |
| AUT | .323 | .030 | .145 | -.030 | -.082 | .403 |  | .223 | .143 | .208 | .081 | .121 | .754 |  |
| SOC | .404 | .006 | -.085 | .019 | .282 | .100 | .313 | .380 | .059 | .130 | .037 | .061 | -.034 | .685 |

**Table S12.**

*Genetic and Environmental Correlations From AE Cholesky Model*.

| **Index** | **rA1** | **rA2** | **rA3** | **rA4** | **rA5** | **rA6** | **rA7** | **rE1** | **rE2** | **rE3** | **rE4** | **rE5** | **rE6** | **rE7** |
| --- | --- | --- | --- | --- | --- | --- | --- | --- | --- | --- | --- | --- | --- | --- |
| LS | 1.000 |  |  |  |  |  |  | 1.000 |  |  |  |  |  |  |
| M | .831 | 1.000 |  |  |  |  |  | .600 | 1.000 |  |  |  |  |  |
| OPT | .686 | .550 | 1.000 |  |  |  |  | .307 | .358 | 1.000 |  |  |  |  |
| ANA | .579 | .551 | .590 | 1.000 |  |  |  | .518 | .426 | .267 | 1.000 |  |  |  |
| PA | .752 | .806 | .734 | .407 | 1.000 |  |  | .516 | .455 | .349 | .358 | 1.000 |  |  |
| AUT | .593 | .523 | .599 | .381 | .495 | 1.000 |  | .266 | .296 | .348 | .264 | .333 | 1.000 |  |
| SOC | .675 | .567 | .359 | .378 | .662 | .414 | 1.000 | .475 | .344 | .312 | .309 | .351 | .156 | 1.000 |

**Table S13.**

*Confidence Intervals for Variance Components (AE Cholesky Model)*.

| **Index score** | **Additive genetic effects [95% CI]** | **Non-shared environmental effects**  **[95% CI]** |
| --- | --- | --- |
| Life satisfaction | .27 [.17, .35] | .73 [.65, .83] |
| Meaning in life | .33 [.23, .42] | .67 [.58, .77] |
| Optimism | .35 [.27, .42] | .65 [.58, .73] |
| Absence of negative affect | .34 [.26, .42] | .66 [.58, .74] |
| Positive affect | .26 [.17, .36] | .74 [.64, .83] |
| Autonomy | .30 [.21, .38] | .70 [.62, .79] |
| Social | .36 [.26, .45] | .64 [.55, .74] |

**R Code**

***Study 1***

### Code for exploratory factor analysis ###

### Manuscript title: "The structure of wellbeing—a single underlying factor with

### genetic and environmental influences"

### Date: 25.04.2023

# Load packages

library(dplyr)

library(magrittr)

library(psych)

library(car)

library(ggcorrplot)

library(haven)

# Read data

wellbeing_data_20 <- read.csv("QoL2020_prepared_hfac.csv")

# Subset dataframe to only include variables which go into the EFA

wb_sub <- wellbeing_data_20 %>% select(c("A1a","C1","C2","O1","A2","A3","A4","A5","A6","C4","C5","C6","C21_rec",

"C22_rec","C23_rec","C24_rec","C25_rec","C7","C8","C9","C12","C13","C15",

"C17","M11","M12", "B1", "B2_rec", "B3_rec", "B4_rec", "B6", "B7", "B8_rec",

"B12_rec","SKH10","SKH11","SKH12"))

wb_sub <- zap_labels(wb_sub)

wb_sub_ren <- rename(wb_sub,"Q21"="A1a","Q22"="C1","Q28"="C2","Q29"="O1",

"Q1"="A2","Q2"="A3","Q3"="A4","Q4"="A5","Q5"="A6",

"Q6"="C4","Q7"="C5","Q8"="C6",

"Q16"="C21_rec","Q17"="C22_rec","Q18"="C23_rec","Q19"="C24_rec","Q20"="C25_rec",

"Q9"="C7","Q10"="C8","Q11"="C9","Q12"="C12","Q13"="C13","Q14"="C15", "Q15"="C17",

"Q26"="M11","Q27"="M12",

"Q23"="B1","Q24"="B2_rec","Q25"="B3_rec",

"Q30"="B4_rec","Q31"="B6","Q32"="B7","Q33"="B8_rec","Q34"="B12_rec",

"Q35"="SKH10","Q36"="SKH11","Q37"="SKH12")

# Create correlation matrix using Spearman's correlation

cormatr <- cor(wb_sub_ren, use = "pairwise", method="spearman")

cormatr_rounded <- round(cormatr, 2)

print(cormatr_rounded)

ggcorrplot(cormatr_rounded, lab = TRUE, hc.order=TRUE, lab_size= 4)

# Conduct Bartlett's test and KMO

cortest.bartlett(wb_sub_ren, n = 17417)

KMO(wb_sub_ren)

# Criteria for retaining factors: Scree plot, PA, MAP, EGA

# Scree plot

scree(cormatr,factors=TRUE,pc=FALSE,hline="-1",main="Scree Plot")

# PA

fa.parallel(cormatr, n.obs=17417, fa="fa",fm="wls")

# PA: With WLS estimator and Pearson correlation (estimated from data): Identical results

fa.parallel(wb_sub_ren, fa="fa",fm="wls",use="pairwise",cor="cor")

# MAP

VSS(cormatr,fm="wls",plot=FALSE,n.obs=17417,n=10,rotate=”promax”)

# MAP: With WLS estimator and Pearson correlation (estimated from data).

# Results: "The Velicer MAP achieves a minimum of 0.01 with 6 factors "

VSS(wb_sub_ren, fm = "wls",plot=TRUE,n=10,rotate="promax")

# Inspect factors 10 to 6 (rotated with promax)

efa_10F_rot <- fa(cormatr, fm='wls', nfactors = 10, n.obs=17417, rotate="promax")

op <- par(mfrow=c(1,1))

corPlot(efa_10F_rot, cex=1.8,main="",show.legend = FALSE,keep.par=FALSE,upper = TRUE,cex.axis = 2)

title("10 Factors with Promax rotation", cex.main = 2)

efa_9F_rot <- fa(cormatr, fm='wls', nfactors = 9, n.obs=17417, rotate="promax")

op <- par(mfrow=c(1,1))

corPlot(efa_9F_rot, cex=1.8,main="",show.legend = FALSE,keep.par=FALSE,upper = TRUE,cex.axis = 2)

title("9 Factors with Promax rotation", cex.main = 2)

efa_8F_rot <- fa(cormatr, fm='wls', nfactors = 8, n.obs=17417, rotate="promax")

op <- par(mfrow=c(1,1))

corPlot(efa_8F_rot, cex=1.8,main="",show.legend = FALSE,keep.par=FALSE,upper = TRUE,cex.axis = 2)

title("8 Factors with Promax rotation", cex.main = 2)

efa_7F_rot <- fa(cormatr, fm='wls', nfactors = 7, n.obs=17417, rotate="promax")

op <- par(mfrow=c(1,1))

corPlot(efa_7F_rot, cex=1.8,main="",show.legend = FALSE,keep.par=FALSE,upper = TRUE,cex.axis = 2)

title("7 Factors with Promax rotation", cex.main = 2)

# Six factor solution: WLS

efa_6F_wls_px <- fa(cormatr, fm='wls', nfactors = 6, rotate="promax", n.obs=17417)

print(efa_6F_wls_px)

print(efa_6F_wls_px$Structure)

# Create factor intercorrelation matrix from rotated 6-factor solution

fact_corr_px=efa_6F_wls_px$Phi

print(fact_corr_px)

op <- par(mfrow=c(1,1))

corPlot(fact_corr_px, cex=1.8,main="",show.legend = FALSE,keep.par=FALSE,upper = TRUE,cex.axis = 2)

title("Factor intercorrelation matrix", cex.main = 2)

# Criteria for retaining higher-order factor(s): Scree plot, PA, MAP, EGA

# Scree

scree(fact_corr_px)

# PA

fa.parallel(fact_corr_px, n.obs=17417, fa="fa",fm="wls", n.iter=500)

# MAP

VSS(fact_corr_px,fm="wls",plot=FALSE,n.obs=17417, n = 3,rotate=”promax”)

# Conduct factor analysis of factor intercorrelation matrix

# Extract 1 higher order factor

efa_1F_ho_px <- fa(fact_corr_px, fm='wls', nfactors = 1, n.obs=17417, rotate = "promax")

print(efa_1F_ho_px)

# Schmid-Leiman Transformation with WLS estimation

SL = schmid(cormatr,nfactors=6,fm="wls",rotate="promax")

print(SL)

## Robustness analyses and Supplementary Plots ##

# Extract factors without promax roation

efa_3F_ho <- fa(fact_corr_px, fm='pa', nfactors = 3, n.obs=17417)

op <- par(mfrow=c(1,1))

corPlot(efa_3F_ho, cex=1.8,main="",show.legend = FALSE,keep.par=FALSE,upper = TRUE,cex.axis = 2)

title("efa 3F_higherorder", cex.main = 2)

efa_2F_ho <- fa(fact_corr_px, fm='pa', nfactors = 2, n.obs=17417)

op <- par(mfrow=c(1,1))

corPlot(efa_2F_ho, cex=1.8,main="",show.legend = FALSE,keep.par=FALSE,upper = TRUE,cex.axis = 2)

title("efa 2F_higherorder", cex.main = 2)

# Descriptives

describe(wb_sub_ren)

# Export supplementary plots

# Correlation matrix

pdf(file = “path and file name”, width = 14, height = 14)

ggcorrplot(cormatr_rounded, lab = TRUE, hc.order=TRUE, lab_size= 4)

dev.off()

# Scree plot (first-order)

pdf(file = “path and file name”, width = 12, height = 12)

scree(cormatr,factors=TRUE,pc=FALSE,hline="-1",main="Scree Plot")

dev.off()

# Parallel analysis (first-order)

pdf(file = “path and file name”, width = 12, height = 12)

fa.parallel(cormatr, n.obs=17417, fa="fa",fm="wls")

dev.off()

# 10 factor structure

pdf(file = “path and file name”, width = 12, height = 18)

op <- par(mfrow=c(1,1))

corPlot(efa_10F_rot, cex=1.8,main="",show.legend = FALSE,keep.par=FALSE,upper = TRUE,cex.axis = 2)

title("10 Factors with Promax rotation", cex.main = 2)

dev.off()

# 9 factor structure

pdf(file = “path and file name ", width = 12, height = 18)

op <- par(mfrow=c(1,1))

corPlot(efa_9F_rot, cex=1.8,main="",show.legend = FALSE,keep.par=FALSE,upper = TRUE,cex.axis = 2)

title("9 Factors with Promax rotation", cex.main = 2)

dev.off()

# 8 factor structure

pdf(file = “path and file name”, width = 12, height = 18)

op <- par(mfrow=c(1,1))

corPlot(efa_8F_rot, cex=1.8,main="",show.legend = FALSE,keep.par=FALSE,upper = TRUE,cex.axis = 2)

title("8 Factors with Promax rotation", cex.main = 2)

dev.off()

# 7 factor structure

pdf(file = “path and file name”, width = 12, height = 18)

op <- par(mfrow=c(1,1))

corPlot(efa_7F_rot, cex=1.8,main="",show.legend = FALSE,keep.par=FALSE,upper = TRUE,cex.axis = 2)

title("7 Factors with Promax rotation", cex.main = 2)

dev.off()

# Scree plot (hierarchical)

pdf(file = “path and file name”, width = 12, height = 12)

scree(fact_corr_px)

dev.off()

# Parallel analysis (hierarchical)

pdf(file = “path and file name”, width = 12, height = 12)

fa.parallel(fact_corr_px, n.obs=17417, fa="fa",fm="wls", n.iter=500)

dev.off()

# Correlation matrix with Pearson's r

cormatr_pearson <- cor(wb_sub_ren, use = "pairwise", method="pearson")

cormatr_pearson <- round(cormatr_pearson, 2)

pdf(file = “path and file name”, width = 14, height = 14)

ggcorrplot(cormatr_pearson, lab = TRUE, hc.order=TRUE, lab_size= 4)

dev.off()

# 6-factor solution with Pearson r and WLS

efa_6F_pears <- fa(cormatr_pearson, fm='wls', nfactors = 6, rotate="promax", n.obs=17417)

pdf(file = “path and file name”, width = 12, height = 18)

op <- par(mfrow=c(1,1))

corPlot(efa_6F_pears, cex=1.8,main="",show.legend = FALSE,keep.par=FALSE,upper = TRUE,cex.axis = 2)

title("6 Factors with Pearson's r matrix and Promax rotation", cex.main = 2)

dev.off()

# 6-factor solution with ML

efa_6F_ml <- fa(cormatr_rounded, fm='ml', nfactors = 6, rotate="promax", n.obs=17417)

pdf(file = “path and file name”, width = 12, height = 18)

op <- par(mfrow=c(1,1))

corPlot(efa_6F_ml, cex=1.8,main="",show.legend = FALSE,keep.par=FALSE,upper = TRUE,cex.axis = 2)

title("6 Factors with ML estimation and Promax rotation", cex.main = 2)

dev.off()

# 6-factor solution with OLS

efa_6F_ols <- fa(cormatr_rounded, fm='ols', nfactors = 6, rotate="promax", n.obs=17417)

pdf(file = “path and file name”, width = 12, height = 18)

op <- par(mfrow=c(1,1))

corPlot(efa_6F_ols, cex=1.8,main="",show.legend = FALSE,keep.par=FALSE,upper = TRUE,cex.axis = 2)

title("6 Factors with OLS estimation and Promax rotation", cex.main = 2)

dev.off()

# 6-factor solution with principal axis

efa_6F_px <- fa(cormatr_rounded, fm='pa', nfactors = 6, rotate="promax", n.obs=17417)

print(efa_6F_px)

print(efa_6F_px$Structure)

pdf(file = “path and file name”, width = 12, height = 18)

op <- par(mfrow=c(1,1))

corPlot(efa_6F_px, cex=1.8,main="",show.legend = FALSE,keep.par=FALSE,upper = TRUE,cex.axis = 2)

title("6 Factors with PA estimation and Promax rotation", cex.main = 2)

dev.off()

# 6-factor solution with PA estimation and oblimin

efa_6F_pa_obl <- fa(cormatr_rounded, fm='pa', nfactors = 6, rotate="oblimin", n.obs=17417)

pdf(file = “path and file name”, width = 12, height = 18)

op <- par(mfrow=c(1,1))

corPlot(efa_6F_pa_obl, cex=1.8,main="",show.legend = FALSE,keep.par=FALSE,upper = TRUE,cex.axis = 2)

title("6 Factors with PA estimation and Oblimin rotation", cex.main = 2)

dev.off()

***Study 2***

### Code for confirmatory factor analysis ###

### Manuscript title: "The structure of wellbeing—a single underlying factor with

### genetic and environmental influences"

### Date: 25.04.2023

# Load packages

library(haven)

library(dplyr)

library(magrittr)

library(psych)

library(car)

library(lavaan)

library(semPlot)

# Load data

data <- read.csv("Hallingdal_prepared.csv")

# Subset data

wellbeing_data <- data %>% select(c("a1a","c1","c2","a2","a3","a4","a5","a6",

"c4","c5","c6",

"c21_rec","c22_rec","c23_rec","c24_rec","c25_rec",

"c7","c8","c9","c12","c13","c15",

"c17","m11","m12","b1","b2_rec","b3_rec","b4_rec","b6","b7",

"b8_rec","b12_rec",

"skh10","skh11","skh12"))

# Rename variables

wellbeing_data_ren <- rename(wellbeing_data,"Q21"="a1a","Q22"="c1","Q28"="c2",

"Q1"="a2","Q2"="a3","Q3"="a4","Q4"="a5","Q5"="a6",

"Q6"="c4","Q7"="c5","Q8"="c6",

"Q16"="c21_rec","Q17"="c22_rec","Q18"="c23_rec","Q19"="c24_rec","Q20"="c25_rec",

"Q9"="c7","Q10"="c8","Q11"="c9","Q12"="c12","Q13"="c13","Q14"="c15", "Q15"="c17",

"Q26"="m11","Q27"="m12","Q23"="b1","Q24"="b2_rec","Q25"="b3_rec","Q30"="b4_rec",

"Q31"="b6","Q32"="b7","Q33"="b8_rec","Q34"="b12_rec",

"Q35"="skh10","Q36"="skh11","Q37"="skh12")

# Primary model: 6F with H and DWLS estimator

CFA_6F_H <- 'LS =~ Q3 + Q1 + Q2 + Q4 + Q5 + Q21 + Q28 + Q22

AUT =~ Q18 + Q17 + Q19 + Q16 + Q20

PA =~ Q7 + Q8 + Q6 + Q31

WF =~ Q13 + Q12 + Q15 + Q14 + Q10 + Q11 + Q9

SOC =~ Q26 + Q27 + Q36 + Q35 + Q37

ANA =~ Q24 + Q33 + Q34 + Q25 + Q30 + Q32 + Q23

H =~ LS + AUT + PA + WF + SOC + ANA'

CFA_6F_H_dwls_fit <- cfa(CFA_6F_H, data = wellbeing_data_ren, estimator="DWLS")

summary(CFA_6F_H_dwls_fit, standardized = TRUE, fit.measures = TRUE, rsquare=TRUE)

# Export plot

pdf(file = "path and file name", width = 9, height = 8)

semPaths(CFA_6F_H_dwls_fit, title=TRUE, rotation=2, style="lisrel", intercepts = TRUE, edge.label.position = 0.6,what="stand",

whatLabels = "std", edge.label.cex=0.55, label.cex=0.8, sizeMan=5, sizeMan2=1.5, edge.color="black",

sizeLat=7, residuals = FALSE, esize =1, asize=2, fade = TRUE, color = "lightgreen",fixedStyle=1)

dev.off()

# Supplementary model b: 6F with H and DWLS estimator excluding skh10 and skh12

CFA_6F_H_b <- 'LS =~ Q3 + Q1 + Q2 + Q4 + Q5 + Q21 + Q28 + Q22

AUT =~ Q18 + Q17 + Q19 + Q16 + Q20

PA =~ Q7 + Q8 + Q6 + Q31

WF =~ Q13 + Q12 + Q15 + Q14 + Q10 + Q11 + Q9

SOC =~ Q26 + Q27 + Q36

ANA =~ Q24 + Q33 + Q34 + Q25 + Q30 + Q32 + Q23

H =~ LS + AUT + PA + WF + SOC + ANA'

CFA_6F_H_b_fit <- cfa(CFA_6F_H_b, data = wellbeing_data_ren, estimator="DWLS")

summary(CFA_6F_H_b_fit, standardized = TRUE, fit.measures = TRUE)

# Plot

pdf(file = "path and file name", width = 9, height = 8)

semPaths(CFA_6F_H_b_fit, title=TRUE, rotation=2, style="lisrel", intercepts = TRUE, edge.label.position = 0.92,

whatLabels = "std", edge.label.cex=0.65, label.cex=0.8, sizeMan=5, sizeMan2=1.5, edge.color="black",

sizeLat=7, residuals = FALSE, esize =1, asize=2, fade = TRUE, color = "lightgreen", fixedStyle=1)

dev.off()

***Study 3.***

# Script for manuscript: "Identifying wellbeing factors and their underlying genetic and environmental architecture"

# Date: 25.04.2023

# Load packages

library(dplyr)

library(haven)

library(car)

library(psych)

library(umx)

library(lavaan)

library(semPlot)

# Reshape to wide format using umx_long2wide

data_v3 <- umx_long2wide(data=data_v2,famID="PairID",twinID="TwinID",zygosity="Groupx",vars2keep = c("ls_std_mean","meaning_std","opt_std_mean",

"aff_rec_std_mean","paff_std_mean","aut_std_mean","soc_std_mean","Sexx","partici2016_1_1987","age2016"))

# Re-format data using umx_mate_twin_data_nice

data_v3 = umx_make_twin_data_nice(data_v3,sep="_T",zygosity="Groupx",levels=1:4,labels=c("MzM","DzM","MzF","DzF"),

labelNumericZygosity = TRUE)

# Mutate so age is co-twin's age if missing and sex is co-twin's sex if missing

data_v4 <- data_v3 %>%

mutate(age2016_T1 = ifelse(is.na(age2016_T1),age2016_T2,age2016_T1)) %>%

mutate(age2016_T2 = ifelse(is.na(age2016_T2),age2016_T1,age2016_T2)) %>%

mutate(Sexx_T1 = ifelse(is.na(Sexx_T1),Sexx_T2,Sexx_T1)) %>%

mutate(Sexx_T2 = ifelse(is.na(Sexx_T2),Sexx_T1,Sexx_T2))

# Residualize all DVs based on age and sex

data_v4_res <- umx_residualize(c("ls_std_mean","meaning_std","opt_std_mean","aff_rec_std_mean",

"paff_std_mean","aut_std_mean","soc_std_mean"),cov = c("age2016","Sexx"),

suffixes = c("_T1","_T2"), data = data_v4)

# Create MZ data and compare numbers to Hoofgard et al. (2021)

mz = data_v4_res %>% filter(zygosity == "MzM" | zygosity == "MzF")

dz = data_v4_res %>% filter(zygosity == "DzM" | zygosity == "DzF")

# Multivariate Cholesky models

selVars <- c("ls_std_mean_T1","ls_std_mean_T2","meaning_std_T1","meaning_std_T2","opt_std_mean_T1","opt_std_mean_T2",

"aff_rec_std_mean_T1","aff_rec_std_mean_T2","paff_std_mean_T1","paff_std_mean_T2",

"aut_std_mean_T1","aut_std_mean_T2","soc_std_mean_T1","soc_std_mean_T2")

mzData <- mz[,selVars]

dzData <- dz[,selVars]

round(cor(mzData, use="pairwise"),3)

round(cor(dzData, use="pairwise"),3)

# Multivariate Cholesky model: ACE

Chol_ACE=umxACE(selDVs = c("ls_std_mean_T","meaning_std_T","opt_std_mean_T","aff_rec_std_mean_T","paff_std_mean_T",

"aut_std_mean_T","soc_std_mean_T"),dzData = dzData,mzData = mzData,sep="")

umxSummary(Chol_ACE,std = TRUE)

plot(Chol_ACE)

parameters(Chol_ACE)

# Multivariate Cholesky model: ADE

Chol_ADE=umxACE(selDVs = c("ls_std_mean_T","meaning_std_T","opt_std_mean_T","aff_rec_std_mean_T","paff_std_mean_T",

"aut_std_mean_T","soc_std_mean_T"),dzData = dzData,mzData = mzData,sep="",dzCr=.25)

umxSummary(Chol_ADE,std = TRUE)

plot(Chol_ADE)

parameters(Chol_ADE)

# Multivariate Cholesky model: AE

Chol_AE = umxModify(Chol_ACE, update = c("c_r1c1","c_r2c1","c_r3c1","c_r4c1","c_r5c1","c_r6c1",

"c_r2c2","c_r3c2","c_r4c2","c_r5c2","c_r6c2",

"c_r3c3","c_r4c3","c_r5c3","c_r6c3",

"c_r4c4", "c_r5c4", "c_r6c4",

"c_r5c5", "c_r6c5",

"c_r6c6",

"c_r7c1","c_r7c2","c_r7c3","c_r7c4","c_r7c5","c_r7c6","c_r7c7"), name = "Cholesky AE",comparison=TRUE)

umxSummary(Chol_AE,std= TRUE,showRg=TRUE, digits=3)

parameters(Chol_AE)

plot(Chol_AE)

# # Multivariate Cholesky model: CE

Chol_CE = umxModify(Chol_ACE, update = c("a_r1c1","a_r2c1","a_r3c1","a_r4c1","a_r5c1","a_r6c1",

"a_r2c2","a_r3c2","a_r4c2","a_r5c2","a_r6c2",

"a_r3c3","a_r4c3","a_r5c3","a_r6c3",

"a_r4c4", "a_r5c4", "a_r6c4",

"a_r5c5", "a_r6c5",

"a_r6c6",

"a_r7c1","a_r7c2","a_r7c3","a_r7c4","a_r7c5","a_r7c6","a_r7c7"),

name = "Cholesky CE",comparison=TRUE)

umxSummary(Chol_CE, std = TRUE)

parameters(Chol_CE)

plot(Chol_CE)

# # Multivariate Cholesky model: E

Chol_E = umxModify(Chol_ACE, update = c("a_r1c1","a_r2c1","a_r3c1","a_r4c1","a_r5c1","a_r6c1",

"a_r2c2","a_r3c2","a_r4c2","a_r5c2","a_r6c2",

"a_r3c3","a_r4c3","a_r5c3","a_r6c3",

"a_r4c4", "a_r5c4", "a_r6c4",

"a_r5c5", "a_r6c5",

"a_r6c6",

"a_r7c1","a_r7c2","a_r7c3","a_r7c4","a_r7c5","a_r7c6","a_r7c7",

"c_r1c1","c_r2c1","c_r3c1","c_r4c1","c_r5c1","c_r6c1",

"c_r2c2","c_r3c2","c_r4c2","c_r5c2","c_r6c2",

"c_r3c3","c_r4c3","c_r5c3","c_r6c3",

"c_r4c4", "c_r5c4", "c_r6c4",

"c_r5c5", "c_r6c5",

"c_r6c6",

"c_r7c1","c_r7c2","c_r7c3","c_r7c4","c_r7c5","c_r7c6","c_r7c7"),

name = "Cholesky E",comparison=TRUE)

umxSummary(Chol_E, std = TRUE)

parameters(Chol_E)

plot(Chol_E)

umxCompare(base=Chol_ACE,comparison=c(Chol_ADE,Chol_AE,Chol_CE,Chol_E),report="inline")

# Common pathway model: ACE (Not reported)

CP_ACE=umxCP(selDVs=c("ls_std_mean_T","meaning_std_T","aff_rec_std_mean_T","paff_std_mean_T",

"opt_std_mean_T","aut_std_mean_T","soc_std_mean_T"),

dzData=dzData, mzData=mzData,sep="",nFac=1)

umxSummary(CP_ACE,std=T)

umxParameters(CP_ACE)

plot(CP_ACE)

# Common Pathway model: AE (Reported in manuscript)

CP_AE <- umxModify(CP_ACE,update=c("c_cp_r1c1",

"cs_r1c1","cs_r2c2","cs_r3c3","cs_r4c4",

"cs_r5c5","cs_r6c6","cs_r7c7"),

comparison=TRUE,name="CP_AE")

umxSummary(CP_AE, std = TRUE)

plot(CP_AE)

umxCompare(base=Chol_ACE,comparison=c(Chol_AE,CP_AE))

# RMSEA estimates: Cholesky models

RMSEA(Chol_ACE)

RMSEA(Chol_ADE)

RMSEA(Chol_AE)

RMSEA(Chol_CE)

RMSEA(Chol_E)

# RMSEA estimates: CP model

RMSEA(CP_AE)

# CFA in twin data

CFA_v1_std = 'LS =~ S_22_1_std + S_22_2_std + S_22_3_std + S_22_4_std + S_22_5_std

M =~ meaning_std

OPT =~ S_31_1_std + S_31_2_rec_std + S_31_3_std + S_31_4_rec_std + S_31_5_rec_std + S_31_6_std

NAFF =~ S_24_1_rec_std + S_24_2_rec_std + S_24_3_rec_std + S_24_4_rec_std + S_24_5_rec_std + S_24_6_rec_std + S_24_7_rec_std + S_24_8_rec_std

PAFF =~ S_25_1_1_std + S_25_1_2_std + S_25_1_3_std

AUT =~ S_26_1_std + S_26_2_std + S_26_3_std + S_26_4_std + S_26_5_std

SOC =~ S_32_1_std + S_32_2_rec_std + S_32_3_std + S_32_4_std + S_32_5_std

H =~ LS + M + OPT + NAFF + PAFF + AUT + SOC'

# Reported in the manuscript: DWLS estimator

CFA_v1_fit_dwls_std <- cfa(CFA_v1_std, data=data,estimator="dwls")

summary(CFA_v1_fit_dwls_std,standardized=TRUE,fit.measures=TRUE)

semPaths(CFA_v1_fit_dwls_std,title=TRUE,rotation=2,style="lisrel",intercepts=TRUE,edge.label.position=0.92,whatLabels="std",edge.label.cex=0.65,

label.cex=0.8,sizeMan = 5,sizeMan2 = 1.5,edge.color = "black",sizeLat=7,residuals=FALSE,esize=1,asize=2,fade=TRUE)

**References**

Diener, E., Emmons, R. A., Larsen, R. J., & Griffin, S. (1985). The Satisfaction With Life Scale. *Journal of Personality Assessment*, *49*(1), 71–75. https://doi.org/10.1207/s15327752jpa4901_13

Izard, C. E., Libero, D. Z., Putnam, P., & Haynes, O. M. (1993). Stability of emotion experiences and their relations to traits of personality. *Journal of Personality and Social Psychology*, *64*(5), 847–860. https://doi.org/10.1037/0022-3514.64.5.847

Leganger, A., Kraft, P., & Røysamb, E. (2000). Perceived self-efficacy in health behaviour research: Conceptualisation, measurement and correlates. *Psychology & Health*, *15*(1), 51–69. https://doi.org/10.1080/08870440008400288

Nilsen, T. S., Brandt, I., Czajkowski, N., Knudsen, G. P., Magnus, P., Reichborn-Kjennerud, T., Tambs, K., Harris, J. R., & Ørstavik, R. (2016). The Norwegian Twin Registry. *Norsk Epidemiologi*, *26*(1–2), 19–27. https://doi.org/10.5324/nje.v26i1-2.2012

OECD. (2013). *OECD Guidelines on Measuring Subjective Well-being*. OECD Publishing. http://dx.doi.org/10.1787/9789264191655-en

Røysamb, E., Vittersø, J., & Tambs, K. (2014). The Relationship Satisfaction scale – Psychometric properties. *Norsk Epidemiologi*, *24*(1–2), 187–194.

Scheier, M. F., Carver, C. S., & Bridges, M. W. (1994). Distinguishing optimism from neuroticism (and trait anxiety, self-mastery, and self-esteem): A reevaluation of the Life Orientation Test. *Journal of Personality and Social Psychology*, *67*(6), 1063–1078. https://doi.org/10.1037/0022-3514.67.6.1063

Tambs, K., & Røysamb, E. (2014). Selection of questions to short-form versions of original psychometric instruments in MoBa. *Norsk Epidemiologi*, *24*(1–2), 195–201.

Watkins, M. W. (2020). *A Step-by-Step Guide to Exploratory Factor Analysis with R and RStudio* (1st ed.). Routledge. https://www.routledge.com/A-Step-by-Step-Guide-to-Exploratory-Factor-Analysis-with-R-and-RStudio/Watkins/p/book/9780367634681
